# Expression of TNF-superfamily members BAFF and APRIL in breast cancer: Immunohistochemical study in 52 invasive ductal breast carcinomas

**DOI:** 10.1186/1471-2407-8-76

**Published:** 2008-03-20

**Authors:** Vassiliki Pelekanou, Marilena Kampa, Maria Kafousi, Katerina Darivianaki, Elias Sanidas, Dimitrios D Tsiftsis, Efstathios N Stathopoulos, Andreas Tsapis, Elias Castanas

**Affiliations:** 1 Laboratory of Experimental Endocrinology, University of Crete, School of Medicine, Heraklion, 71003, Greece; 2 Department of Pathology, University of Crete, School of Medicine, Heraklion, 71003, Greece; 3 Department of Surgical Oncology, University of Crete, School of Medicine, Heraklion, 71003, Greece; 4 INSERM, Unité 841, Faculté de Medécine, Université de Paris 12, Créteil, 94010, France

## Abstract

**Background:**

Recent studies suggest an association between chronic inflammation, modulating the tissue microenvironment, and tumor biology. Tumor environment consists of tumor, stromal and endothelial cells and infiltrating macrophages, T lymphocytes, and dendritic cells, producing an array of cytokines, chemokines and growth factors, accounting for a complex cell interaction and regulation of differentiation, activation, function and survival of tumor and surrounding cells, responsible for tumor progression and spreading or induction of antitumor immune responses and rejection. Tumor Necrosis Factor (TNF) family members (19 ligands and 29 receptors) represent a pleiotropic family of agents, related to a plethora of cellular events from proliferation and differentiation to apoptosis and tumor reduction. Among these members, BAFF and APRIL (CD257 and CD256 respectively) gained an increased interest, in view of their role in cell protection, differentiation and growth, in a number of lymphocyte, epithelial and mesenchymal structures.

**Methods:**

We have assayed by immunohistochemistry 52 human breast cancer biopsies for the expression of BAFF and APRIL and correlated our findings with clinicopathological data and the evolution of the disease.

**Results:**

BAFF was ubiquitely expressed in breast carcinoma cells, DCIS, normal-appearing glands and ducts and peritumoral adipocytes. In contrast, APRIL immunoreactive expression was higher in non-malignant as compared to malignant breast structures. APRIL but not BAFF immunoreactivity was higher in N+ tumors, and was inversely related with the grade of the tumors. Neither parameter was related to DFS or the OS of patients.

**Conclusion:**

Our data show, for the first time, an autocrine secretion of BAFF and APRIL from breast cancer cells, offering new perspectives for their role in neoplastic and normal breast cell biology and offering new perspectives for possible selective intervention in breast cancer.

## Background

Cancer is a hyperproliferative disorder that involves morphological cellular transformation, dysregulation of apoptosis, uncontrolled cellular proliferation, invasion, angiogenesis, and metastasis [1]. Clinical and epidemiologic studies suggest a strong association between chronic inflammation and tumor initiation, promotion and progression [see [2], for a review]. Recent data from mouse models of human cancer have established that inflammation, orchestrating the tumor microenvironment, is a critical component of both tumor promotion and progression [3,4]. Indeed, the tumor microenvironment consists of a variable combination of tumor cells, stromal fibroblasts, endothelial cells and infiltrating leukocytes, such as macrophages, T lymphocytes, and dendritic cells. A variety of cytokines, chemokines and growth factors are produced in the local tumor environment by different cells accounting for a complex cell interaction and regulation of differentiation, activation, function and survival of multiple cell types. The interaction between cytokines, chemokines, growth factors and their receptors forms a comprehensive network at the tumor site, which is primary responsible for overall tumor progression and spreading or induction of antitumor immune responses and tumor rejection [5].

Current data support the notion that inflammation triggered by tumor-infiltrating host leukocytes does not always exert normal immunoprotective mechanisms which could lead to eradication of the evolving cancer (antitumor immunity). Instead, excessively and chronically produced proinflammatory mediators are thought to contribute to tumor promotion and progression [2,3,6,7], as, in the tumor microenvironment, a delicate balance occurs between antitumor immunity and tumor-originated proinflammatory activity [6,8]. These activities depend on different mediators that are released by host inflammatory cells, cancer cells, and other types of tumor-associated host cells (such as fibroblasts and endothelial cells). When host-mediated antitumor activity is weaker than tumor-mediated immunosuppressive activity, tumor cells undergo immune escape and grow rapidly [9]. By contrast, when host-mediated antitumor immunity is stronger than tumor-mediated immunosuppressive activity, tumor cells are eliminated [9]. The net outcome of a persistent inflammatory microenvironment is enhanced tumor promotion, accelerated tumor progression, invasion of the surrounding tissues, angiogenesis, and often metastasis [5].

Several pro-inflammatory cytokines are known to promote tumor growth, such as tumor necrosis factor -1) (TNFα), interleukin 1 (IL-1), interleukin 6 (IL-6) or , interleukin 8 (IL-8) [4,10]. In addition, a link between activating mutations in oncogenes and inflammation has been recently reported, as activation of *Ras *proto-oncogenes in cancer results in up-regulation of the inflammatory cytokine IL-8, which, in turn, acts as a chemokine and in turn promotes tumor associated inflammation, angiogenesis and eventually tumor growth [11]. The tumor necrosis factor alpha (TNFα) system is of primary importance in the modulation of the immune response, supporting the innate immune response by promoting cell stimulation and pro-inflammatory cytokine secretion. The TNF superfamily (TNF-SF) consists of 19 ligands and 29 receptors and orchestrates a wide range of biological functions, from the regulation of activation and cell death in the immune system to tissue homeostasis and cancer cell modulation [12]. Recently two new TNF ligands were discovered through expressed sequence tag (EST) database searches: a proliferation-inducing ligand (APRIL or CD256, TNFSF13) [13-15] and B lymphocyte stimulator (BLyS), which is also known as B-cell activating factor of the TNF family (BAFF, also reported as TALL-1, CD257 and TNFSF13B) [15-18]. Both ligands bind to two TNF-R family members, transmembrane activator and CAML interactor (TACI) and B-cell maturation antigen (BCMA [19], reviewed in [20]). BAFF also specifically binds to another TNF-R family member, BAFF-R. The interaction pattern between BAFF, APRIL and their receptors is both specific and redundant: Binding to their respective receptors leads to the triggering of diverse signaling pathways, including the activation of caspases, the translocation of nuclear factor kappaB (NFkappaB), or the activation of mitogen-activated kinases such as c-Jun NH2-terminal kinase (JNK) or extracellular signal regulatory kinase (ERK) [21], (see [22], for a review). BAFF and APRIL were recently found to be trophic factors in lymphocyte malignancies and immune-related disorders [23], while they have been identified in bronchial tissue [24], and a number of immune-rerated and immune-independent tissues (spleen, liver, lung, heart, intestine, kidney, thymus) [25]. Interestingly, a recent report indicates further that activation of this system promotes the proliferation of glioblastoma cell lines [26], while the origin of these factors in extra-hematologic malignancies is not fully established. Indeed, both BAFF and APRIL could be synthesized and secreted by B- or T-tumor infiltrating lymphocytes, dendritic cells or other components of the tumor microenvironment [27]. In the present report, we provide, for the first time, evidence indicating the production of these two trophic factors by epithelial breast cancer cells, indicative for a supplementary autocrine function of cancer cells. We further report that BAFF is expressed in a constant way by malignant and peritumoral non-malignant epithelial cells, while the production of APRIL is high in non-malignant and decreased in malignant breast carcinoma cells. In this respect, our data are opposing from the current dogma of increased APRIL production in malignancies, suggesting a possible new role of these two TNF-SF members in epithelial malignancies and provide additional elements of cell growth regulation of breast cancer.

## Methods

### Patients

Fifty two (52) patients, operated for ductal breast cancer in the Surgical Oncology Department of the University Hospital of Heraklion, have been included in the present study. Patients were followed from 2–70 months (overall survival, median 46 months, mean 44.8 months). Disease-free survival ranged from 1–69 months (median 46 months, mean 41.5 months). All patients have followed a pre-operative and post-operative chemotherapy and radiotherapy and 40 had an adjuvant hormone therapy. Thirteen patients were staged as T_1_N_1_M_0_, 28 patients were T_2_N_1_M_0_, while the stage of the remaining patients ranged from T_1_N_0_M_0 _to T_3_N_1_M_0_, according to the tumor size (T), existence of positive node (N) or distal metastases (M, each parameter staging from 0 to 3) (Table 1) [28]. An informed consent was signed by all patients before inclusion in the present study. The Ethics and Scientific committees of the University Hospital have approved the present study.

**Table 1:** Clinicopathological characteristics of patients included in the present study

	Mean	Median	Minimum	Maximum	No of Cases
Age	56.24	58	26	77	
Mean Diameter	3.0	2.5	0.8	9.5	
Grade		2	1	3	
DFS (months)	41.51	46	1	69	
OS (months)	44.8	46	2	69	
TNM Staging					
T1N0M0					3
T1N1M0					13
T2N0M0					2
T2N1M0					28
T3N1M0					4
T4N1M0					2

### Tumor analysis

All tumor specimens were formalin-fixed and paraffin-embedded. They were analyzed at the Pathology Department of the University of Crete. Three serial sections (3μm) were cut from each tissue-block. One was stained with hematoxylin and eosin and two sequential slides were used for the immunohistochemical detection of APRIL and BAFF. The slides were reviewed by two investigators independently and blindly to the rest clinicopathologic data, and the final conclusion was reached in consensus. In few cases with discrepancy, between the two observers, greater than 10%, the immunostained slides were reviewed in a double viewing microscope so that the discrepancy was settled. Histopathological and clinical data were retrieved from the Pathology and Surgical Oncology Department databases, including size and grade of the tumor, TNM status, overall and disease-free survival and treatment. These data are summarized in Table 1.

### Immunohistochemical Staining for APRIL and BAFF

**Figure 1:**
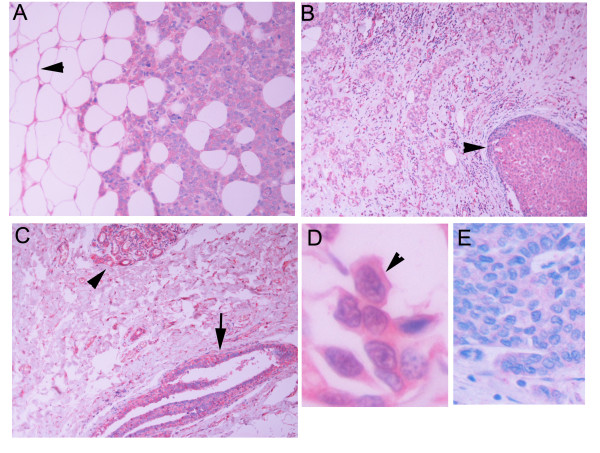
BAFF immunoreactivity in breast carcinoma (A), DCIS (B, arrowhead) and normal appearing duct (C, arrow) and lobule (C, arrowhead). Normal adipocytes are stained positively for BAFF (Pannel A, arrowhead). In D, a higher magnification is shown, in which a homogeneous cytoplasmic BAFF immunoreactivity is shown, with more prominent cell membrane staining. E: Normal Ig isotype staining.

**Figure 2:**
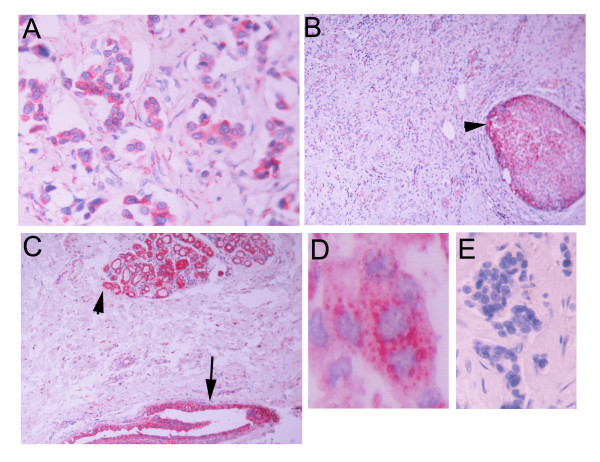
APRIL immunoreactivity in invasive breast carcinoma (A), DCIS component of the same carcinoma (B, arrowhead), and normal looking ducts (C, arrow) and lobules (C, arrowhead). In D, a higher magnification is shown. APRIL immunoreactivity is presented as discrete cytoplasmic dots. E: Normal Ig isotype staining.

After deparaffinization and hydration, slides were subjected to three cycles (5 min) of citrate buffer (0.01 M, pH 6.0) incubation in a microwave oven (500 W), and treated with 3% hydrogen peroxide for 15 min. They were then incubated with primary antibodies for APRIL (hAprily-8 mouse monoclonal antibody, ALX-804-149, Alexis Co, Lausen, Switzerland) in a dilution of 1/100, or BAFF (804-131-C100 monoclonal antibody/Buffy-2 clone, Alexis Co, Lausen, Switzerland) in a dilution of 1/200. The UltraVision LP Detection System (TL-060-AL, Lab Vision Co, Fremont, USA) with Fast Red as chromogen was used for immunodetection. Counterstaining was performed using controls (omission of the primary antibody) were used in every run, while a rat or mouse mAB isotype (see Figures 1E and 2E respectively) confirmed the specificity of staining.

The tumor sections and tissues in their neighbourhood, included in each one of the whole sections embedded, were examined and the following components were recorded: representative areas of the tumor (excluding necrotic regions), the tumor growing edge, and extratumoral structures when present in the tissue block examined; namely elements of *in situ *ductal carcinoma (DCIS) at the vicinity of the invasive component of the carcinoma, ducts of the mammary tissue with hyperplasia, normal ducts and lobules The results of the study of each component studied were reported as a percentage of stained cells at a given intensity, graded in a scale of 1–3. The H-score [29] was used for the analysis of results, calculating the intensity and the percentage of staining by the formula %*1+ %*2+%*3.

### Immunohistochemical staining of BCMA, TACI and BAFF-R

After deparaffinization and hydration, slides were subjected to three cycles (5 min) of citrate buffer (0.01 M, pH 6.0) incubation in a microwave oven (500 W), and treated with 3% hydrogen peroxide for 15 min. They were then incubated with primary antibodies for BCMA (Vicky-1 rat monoclonal antibody, ALX-804-151, Alexis Co, Lausanne, Switzerland) in a dilution of 1/100, for TACI (IMG-249 rabbit polyclonal antibody, Imgenex, San Diego, CA, USA) in a dilution of 1/100 and for BAFF-R (AF1162, goat polyclonal antibody, R&D Systems, Minneapolis, MN, USA). The UltraVision LP Detection System (TL-060-AL, Lab Vision Co, Fremont, USA) with Fast Red as chromogen was used for immunodetection. Counterstaining was performed using Mayer's hematoxylin. Known positive and negative controls (omission of the primary antibody) were used in every run.

### Statistical Analysis

Statistical analysis was performed by the use of appropriate parametric and non-parametric tests, as described in the Results section, by the use of SPSS v 14 and AMOS v6 (SPSS Inc, Chicago, IL). The statistical significance was settled at 0.05.

## Results

### Immunohistochemical detection of BAFF and APRIL in breast cancer specimens

In the tumor specimens examined, we have further identified 24 cases with DCIS component, 17 cases with hyperplastic ducts in the vicinity of the carcinoma, and 9 cases with normal lobules in the neighbourhood of the main lesion. BAFF was positive in all but one cases of cancer, with an H-score ranging from 30–288 (mean = 145, median 147), in all cases of DCIS (mean H-score = 163, median 184, range 44–200), in all assayed cases of ducts (range 145–300) and in all three assayed cases of normal lobules (H-score range 140–200).

Typical cases are presented in Figure 1. As shown, BAFF immunoreactivity was detected in the cytoplasm, wherein some cases a more intense perinuclear staining was observed. Finally, the periphery of the cells was more heavily stained. This result is explained by the fact that, although BAFF is a secreted protein, anchored in the cell membrane, the thin histological sectioning of our specimens (3 μm) results in an exposure of the BAFF-synthesizing cytosol to the antibody, expressing an intracellular staining. Interestingly, peritumoral adipose tissue was also positive for BAFF staining. Adipocyte cytoplasm and membranes were always stained for BAFF, while lipid droplet was negative.

APRIL staining on the other hand, was positive in 39/53 cases of cancer (mean H-score = 60, median = 15, range 2–282), in 13/15 cases of DCIS (mean H-score = 92, median = 64, range 6–255), in 7/8 cases of normal ducts (mean H-score 215, range 110–300) and in 5/5 normal lobules (mean H-score = 171, range 56–300). In contrast to BAFF, APRIL immunostaining was restricted in well described intracellular dots in specific cytoplasmic areas (Figure 2).

**Figure 3:**
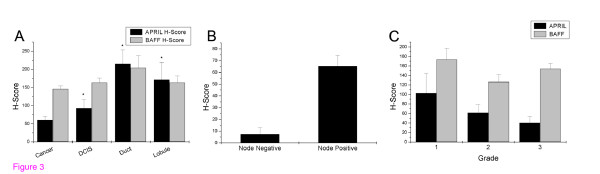
**A**. Distribution of APRIL (black bars) and BAFF (gray bars) immunoreactivity in different areas of breast cancer specimens. The intensity of staining was determined by the use of H-score, as described in the Material and Methods section. Mean ± SEM is presented. Asterisk indicates significantly different results (p < 0.05 at least) as determined by ANOVA with post-hoc mean comparison, after application of the Bonferroni correction. **B**. Distribution of APRIL immunoreactivity in node-negative (n = 4) and node-positive (n = 35) patients. Mean ± SEM is presented. **C**. Distribution of APRIL and BAFF immunoreactivity according to the Grade of patients. Mean ± SEM is presented.

Distribution of BAFF-APRIL staining intensity by site of detection is presented in Figure 3. As shown, BAFF was equally distributed in cancer and non-cancer sites, suggesting a possible trophic role of the agent in breast tissue. In contrast, APRIL expression is low in cancer, mediately elevated in DCIS while it attains its maximum in non-cancer breast tissue. This result indicates a differential role of the two ligands in breast cancer disease and is supported by the significant dependence of APRIL expression to the site of detection (p < 0.0001 in both cases, ANOVA with the Bonferroni correction).

### Detection of BAFF-R, BCMA and TACI in breast cancer specimens

BAFF and APRIL bind to three different receptors: BAFF-R, TACI and BCMA [20]. We have assayed these three receptors by immunohistochemistry in subsequent sections on the same cancer cases. In all cases, the three receptors were negative (not shown).

### Correlation of BAFF and APRIL immunoreactivity with clinical data and outcome

As shown in Figure 3A, APRIL immunoreactivity decreases as normal breast ducts and lobules progress to cancer. However, a detailed analysis of cancer cases, presented in Figure 3C reveals a negative, although not significant, relationship of APRIL immunostaining with the grade of the tumor (H-score ranging from 102 to 40, Figure 3C). When cases were stratified according to the existence of lymph-node metastases, a significant higher APRIL-H-score was found in N-positive, as compared to N-negative tumors (H-scores 7.4 ± 5.7 and 65.4 ± 12.0, mean ± SEM for N_0 _and N_1 _tumors respectively, t = 4.37, p < 0.0001). In contrast, no such a trend is observed in BAFF-immunostaining. No relationship of APRIL- or BAFF-immunostaining was found however with the DFS or the OS of patients.

## Discussion

Current data relay (chronic) inflammation and cancer, through a complex interplay of autocrine and/or paracrine interactions of signaling molecules (see [5], for a recent discussion). Different cytokines, growth and differentiation factors orchestrate both positive and negative signals for tumor growth, promotion and progression, angiogenesis and metastasis, secreted by either tumor cells themselves, stroma cells or infiltrating populations of lymphocytes and dendritic cells. Among these molecules, TNF-SF ligands and their cognitive receptors play a primordial role. The present work reports, for the first time, the identification of BAFF and APRIL, two members of the TNF-SF, in breast cancer specimens.

TNF ligand family members are usually synthesized as type II transmembrane proteins; cleavage is frequently observed at the plasma membrane. Cleavage usually occurs in the stalk region between transmembrane and receptor-binding domains. In contrast to this processing, APRIL is cleaved intracellularly, which leads to its secretion and not to a membrane-bound form [12,30]. Cleavage occurs in the Golgi; blockage of protein transport from the endoplasmic reticulum to the Golgi apparatus abrogated APRIL processing, whereas inhibition of post-Golgi transport, does not interfere with APRIL cleavage, but block secretion of processed APRIL [30]. APRIL processing is mediated at an arginine-rich motif by furin, a ubiquitously expressed pro-protein convertase that processes many inactive precursors including hormones, growth factors and receptors [31]. BAFF and TWEAK, two other TNF-SF members, are also cleaved proteolytically at a multibasic motif, probably by furin [17,32]. In contrast to APRIL, however, these ligands are not processed intracellularly, but are instead released from the cell surface, where they appear as membrane-anchored proteins [33,34]. Data of the present study are consistent with this synthetic-secretory pathway. As presented in Figures 1 and 2, APRIL immunoreactivity is presented as discrete intracellular dots, consistent with vesicle-sequestrated or Golgi-related ligand, while BAFF immunoreactivity is diffuse in the (BAFF-synthesizing) cytoplasm and highly concentrated at the membrane level.

Most, if not all of the members of the TNF receptor family activate NFkB and AP-1 via TRAF family of adaptors [35,36]. However, specific receptors for BAFF and APRIL (BCMA, TACI, BAFF-R) were not identified in breast cancer specimens. Nevertheless, recent reports suggest that APRIL can additionally bind to heparan sulphate proteoglycans (HSPG) on tumor cell surface and initiate tumor growth [37]. It is interesting to note that this binding occurs through another part of APRIL molecule, different from its binding domain, thus permitting the combined binding to both TACI/BCMA and HSPG. HSPGs play an important role in a wide variety of biological responses and processes such as adhesion, migration, proliferation, embryonal development, differentiation, morphogenesis, angiogenesis and blood coagulation [38-40], thus linking APRIL effects to tumor progression. Indeed, preliminary data indicate that APRIL could enhance migration of breast cancer cell lines, in vitro. However, there is no currently established opinion regarding the functional role of APRIL-proteoglycan interaction: Some authors suggest that only a ligand oligomerization occurs after APRIL binding to these membrane structures, facilitating their interaction with cognitive receptors [41] while others oppose a discrete receptor-initiated action after such a binding [37], or a co-receptor activating function of the APRIL-HSPG dimer [42] or HSPG themselves [43].

Until now, very scarce data exist on the identification of the BAFF/APRIL system in breast carcinoma. Hahne et al [13] reported that APRIL could not elicit any proliferative effect on MCF7 breast cancer cells, in contrast to other cancer cell lines from different tissues, providing a hint for the decreased expression of APRIL in breast tumor specimens, reported here. Interestingly, APRIL expression in a number of solid tumors was attributed to infiltrating lymphocytes, while scare tumor types, especially cutaneous carcinomas, overexpressed the ligand [44]. It is interesting to note that in the same study 2159 tumors of different origins (including 130 breast carcinomas) have been assayed. Only 20 tumors expressed APRIL, in the absence of stroma staining, while 50 tumor stroma expressed exclusively APRIL, in the absence of epithelial staining. The authors suggest that APRIL expression in solid tumors might be a result of a response to an exogenous factor (stimulus) than an intrinsic property of the tumor. However, taking into account the results of previous [44] and of the present study (absence of BAFFR, BCMA and TACI), a possible interaction with heparin-sulphate proteoglycans may be suggested. In this context, as discussed previously [44], APRIL-HSPG interaction may represent a paracrine growth-stimulating function, inducing tumor growth [44,45].

Previous data indicate that APRIL, in contrast to many other TNF-related ligands, is a factor promoting survival of tumor cells in tissue culture and when human tumors are transplanted into immune-deficient mice [13]. Indeed, the expression of APRIL was reported to be nearly undetectable in normal tissue but dramatically elevated in tumors. Addition of recombinant APRIL or transfection of tumor lines with APRIL provided a significant growth advantage to lymphoid and nonlymphoid cells [46]. This suggests that APRIL expression may be a consequence of the stress of malignant transformation [27,44]. Our data show, however, that this general concept does not apply to the breast. Indeed, we show for the first time, a direct production of APRIL from the breast non-tumoral epithelium, inversely related to the malignant transformation of cells, suggesting a possible role of APRIL in well differentiated mammary cells and a decline of its production during malignant transformation. This is further verified, as a negative (although not-significant) trend of APRIL immunoreactivity with the grade of the tumor is also found. Alternatively, if the general concept of APRIL up-regulation as a consequence of stress applies equally in the breast, overproduction of the ligand by peritumoral cells might be due to the physical and/or humoral stress exerted on normal cells by the growing tumor. It might therefore be interesting to follow the evolution of APRIL production in different breast carcinoma cells, representing different evolutionary stages of breast cancer and its modulation with (chemo)therapy or under the action of factors affecting breast cell biology. Such an investigation is currently under progress. In contrast to APRIL, we report for the first time that BAFF is ubiquitously expressed in breast carcinomas, DCIS, normal ducts and lobules with the same intensity, indicating a possible trophic effect of the agent in breast tissue. Alternatively, BAFF could be a constitutively expressed protein of the breast epithelial tissues and derived carcinomas.

Another interesting finding of our study is that BAFF is also constitutively expressed by adipocytes surrounding the tumor lesions. Adipose tissue has long been regarded as an almost resting tissue, dedicated solely to energy storage and release. However, in recent years, this view has changed dramatically following new insights into the metabolic and immunological functions of preadipocytes and adipocytes. Indeed, these cells are potent producers of proinflammatory cytokines (such as IL-6 and TNF) and chemokines. Furthermore, adipocytes secrete high amounts of adipokines, such as leptin, adiponectin and resistin, regulating monocyte/macrophage function, and molecules associated with the innate immune system, such as the C1qTNF-related protein superfamily. Finally, preadipocytes and adipocytes express a broad spectrum of functional Toll-like receptors and can be converted into macrophage-like cells [reviewed in [47]]. These data clearly establish the role of adipose tissue as a new member of the immune system. Adipocytes were found to secrete and be sensible in an autocrine way to TNF, via TNFR1 activation [48]. The role of the cytokine was to suppress adipogenesis and to induce apoptosis [49]. Here, however, we show that peritumoral adipocytes synthesize in a constitutive way BAFF, a ligand which, upon binding to its cognitive receptor, induces survival and differentiation of other cell lineages [35], supporting a possible new trophic role of breast adipocytes in the development of breast epithelium. It might therefore be interesting to investigate under a new light the functional interplay of both the BAFF/APRIL system expression and biological functions in adipose tissue proliferation and survival and the role of adipocytes in mammary gland development, function and malignant transformation.

APRIL and BAFF immunoreactivity does not seem to derive from inflammatory cell infiltration of the tumor. Indeed, as presented, no lymphocyte infiltration of tumors was evident in our specimens. It might therefore be attributed to an auto/paracrine secretion of these agents by the breast tissue itself. In addition this immunoreactivity is not related to the outcome of breast cancer patients. Indeed, no significant relationship between BAFF or APRIL H-scores with overall- or disease-free survival of patients was detected. However, APRIL immunoreactivity was higher in tumors metastasized to lymph nodes, as compared to non-metastasized cases. It is not obvious however, whether this is a causal effect of APRIL, being a lymphocyte proliferation factor [35], or a consequence of metastasized tumors.

## Conclusion

The arising question, in view of our data, is which might be the functional role of the TNF-related system of BAFF-APRIL in breast cancer. The rarity of reported results on their role in epithelial cancers and especially in breast carcinoma does not allow us to proceed to an established evaluation of the system in cancer biology. Indeed, BAFF and APRIL may act as tumor promoters in different systems they have been examined. Our results, however, indicate that the constant expression of BAFF, in adipocytes, normal breast cells and their cancer counterparts, might be relevant of the trophic potency in breast, promoting the proliferation and development of normal and malignant breast tissue. In contrast, the decreased expression of APRIL in breast cancer, as compared to non-cancer breast structures, suggests a possible negative role of this factor in breast neoplasia progression. Our data provide new hints about the possible role of BAFF/APRIL in normal and neoplastic breast tissue and possibly other epithelial or mesenchymal structures. They further offer new perspectives on their possible application as new targets for selective therapeutic intervention.

## Abbreviations

APRIL: A Proliferation-Inducing Ligand, BAFF: B-cell Activating Factor, BAFF-R: BAFF Receptor, BCMA: B-cell Maturation Antigen, DCIS: Ductal Carcinoma In Situ, HSPG: Heparan-sulphate proteoglycans, TACI: Transmembrane Activator and CAML Interactor, TNF: Tumor Necrosis Factor, TNF-SF: TNF Superfamily, TNM: Tumor-Node-Metastasis staging, TWEAK:TNF-related weak inducer of apoptosis

## Competing interests

The author(s) declare that they have no competing interests.

## Authors' contributions

VP, MK, MK and KD performed the research. ES and DDT provided clinical data of patients. ENS supervised immunochemical staining and pathology analysis of data. AT, ENS and EC designed and supervised the project. All authors read and approved the final manuscript.

## Pre-publication history

The pre-publication history for this paper can be accessed here:


